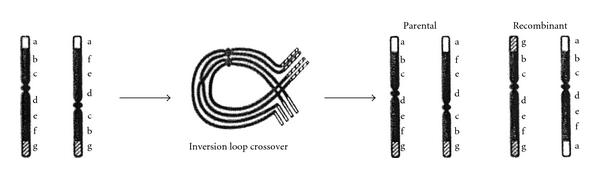# 22q13.32 Deletion and Duplication and Inversion in the Same Family: A Rare Occurrence

**DOI:** 10.5402/2011/829825

**Published:** 2011-06-21

**Authors:** Farooqua Jafri, James Fink, Rodney R. Higgins, Raymond Tervo

**Affiliations:** ^1^Gillette Children's Specialty Healthcare, St. Paul, MN, USA; ^2^Hennepin County Medical Center, Minneapolis, MN, USA; ^3^Abbott Northwestern Hospital, Minneapolis, MN, USA; ^4^Department of Pediatrics, University of Minnesota, Minneapolis, MN, USA

## Abstract

Chromosome 22q13.3 deletion syndrome is a well-recognized cause of global developmental delay, while duplication of the same chromosome is a rare occurrence. The presence of both abnormalities in the same family has never been reported, to our knowledge. We report a rare occurrence of 22q13.3 duplication and 22q13.3 deletion in siblings, as a consequence of a mother's inversion on her 22nd chromosome (p13;q13.32). A 6 year old male was noted in infancy to have mild global developmental delay without dysmorphic features. His genetic testing revealed he had 22q13.3 duplication to the terminus. His 4 year old brother was noted in early infancy to have severe global developmental delay and dysmorphic features related to 22q13.3 deletion to the terminus. Their mother had a long inversion on her 22nd chromosome. Genetic tests for their father and eldest brother were unremarkable. The mother's inversion may rearrange to form 22q duplication or deletion when passed on to children. The chance of a child born with a chromosome imbalance is as high as 50%.

## 1. Introduction

Chromosome 22q13.3 deletion syndrome is a well-recognized cause of global developmental delay (GDD) [1, 2] while duplication of the same chromosome is a rare occurrence [3–5]. The presence of both abnormalities in the same family has never been reported, to our knowledge. We report the presence of 22q13.3 deletion and 22q13.3 duplication in two siblings, with the mother carrying an inversion of chromosome 22 (46, XX, inv (22) (p13q13.32)) A 6 year-old male was noted in infancy to have mild global developmental delay without dysmorphic features. His genetic testing revealed he had 22q13.3 duplication to the terminus. His 4 year-old brother was noted in early infancy to have severe global developmental delay and dysmorphic features related to 22q13.3 deletion to the terminus. Their mother had a long inversion on her 22nd chromosome. Genetic tests for their father and eldest brother were unremarkable. The mother's inversion may rearrange to form 22q duplication or deletion when passed on to children. The chance of a child born with a chromosome imbalance is as high as 50%.

## 2. Case Report

### 2.1. Patient 1

CC was born to a 35-year-old female after a term gestation. His birth weight was 8 lb. 12 oz., and he was delivered by vacuum extraction. His APGAR scores at 1 and 5 minutes were 9 and 10, respectively. He had calcaneo-valgus deformity of the right foot which improved with manipulation with each diaper change.

At 9-month-old, his mother noticed that he would not engage with her and would stare into space. He was referred to a neurodevelopmental pediatrician at age 18 months because all developmental milestones were delayed. His height and weight were at 50th percentile, and fronto-occipital circumference (FOC) was at 98th percentile (large head). No neurocutaneous abnormalities were seen. He had no obvious dysmorphic features. He had a single palmar crease bilaterally, and all joints were hypermobile.

Neuropsychological evaluation revealed significant difficulty in social skills and adaptive functioning, consistent with a diagnosis of “Pervasive Developmental Disorder NOS”. It further revealed that receptive language was better than expressive language skills.

A metabolic disorder screen (plasma for amino acids, urine for organic acids, Smith-Lemli Opitz, thyroid) and Fragile-X screening were normal. Visual and hearing acuity was within normal limits. Echocardiogram, renal ultrasound, and cerebral MRI scan were normal.

**Figure 1:**
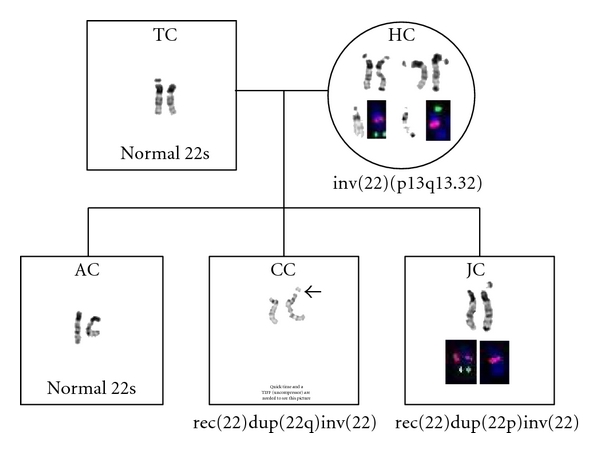
Meiotic pairing in a pericentric inversion heterozygote. An inversion loop containing a single crossover and the resulting parental and recombinant chromosomes. Initials indicate which recombinant/parental chromosome each sibling received from the heterozygote mother (HC) [6].

**Figure 2:**
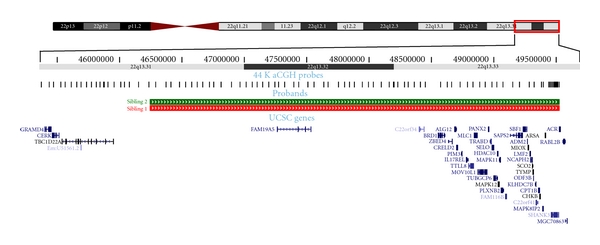


Chromosomal analysis revealed 46, XY, rec (22) dup (22q) inv (22) (p13; q13.32) mat (Figures 1 and 2).

### 2.2. Patient 2

JC is the younger sibling who was born by normal vaginal delivery, after a term gestation, when his mother was 38 yrs old. An amniocentesis in the early part of pregnancy was unrevealing. His birth weight was 9 lb. 3 oz. He had transient tachypnea of the newborn which responded to oxygen administration. There was no cyanosis. An echocardiogram revealed a patent ductus arteriosus, which soon resolved. He also developed hyperbilirubinemia which reversed with phototherapy. He was discharged on day 3.

JC was referred to a neurodevelopmental pediatrician at the age of 13 mos. because of delayed language and motor development. All his developmental domains were delayed.

Height and weight were in the 90th percentile, and FOC was between 70 and 90th percentile. No neurocutaneous abnormalities were seen. There was mixed hearing loss, bilaterally. He had downslanting palpebral fissures, fleshy hands, dysplastic toe nails, and clinodactyly. He has generalized coarse facial features, broad nasal bridge, relatively flat midface, and prominent chin and ears. He has heat intolerance (because of decreased perspiration), a high pain threshold, and a wobbly gait (because of hypotonia).

Behavior characteristics included mouthing and chewing of nonfood items and autistic-like behavior and affect, typical of the 22q13.3 deletion syndrome. Neuropsychological evaluation showed pervasive developmental and ADHD problems. Major behavioral concerns were aggressiveness with biting and hitting caregivers and pets. There was no evidence of self-injurious behavior. 

Newborn genetic screening (amino academia, congenital adrenal hyperplasia, congenital hypothyroidism, Smith-Lemli Opitz, galactosemia, hemoglobinopathy) was normal.

Renal ultrasound and voiding cystourethrogram (VCUG) were normal. Cerebral MRI scan was normal.

Chromosomal analysis revealed 46, XY, rec (22) dup (22p) inv (22) (p13q13.32) mat (Figures 1 and 2). 

Medication management for his disruptive behaviors was strongly recommended, but the family refused this option because of the potential sideeffects.

### 2.3. Parents HC and TC

The mother (HC) has an inversion in one of her number 22 chromosomes: 46, XX, inv(22) (p13; q13.32)—indicating that one of the mother's number 22 chromosomes contains a large piece that is inverted or flipped backwards (Figure 3). HC is phenotypically normal and has no dysmorphic symptoms. She had 2 miscarriages in the past. Her mother had several miscarriages. Her mother's first paternal cousin has an intellectual disability but went on to have 3 children, and one of them is intellectually disabled.

The father (TC) has entirely normal chromosomes. The oldest child has no chromosomal abnormalities.

## 3. Discussion

22q13 would not be considered a classic “hot spot” for genomic rearrangements, which are generally regions flanked by highly homologous segmental duplications which predispose to nonhomologous allelic recombination during meiosis in one of the parental lines, yielding identical and recurrent deletions or the reciprocal duplication.

Pericentric inversions are balanced rearrangements that do not affect the phenotype of the carrier [7]. Chromosome 22 inversion is a rare occurrence (0.01%) [8]. Although such individuals are completely healthy, a certain proportion of their gametes will contain a rearranged form of the inverted 22 chromosome. A parent with an inversion or a balanced translocation could transmit the unbalanced form of the translocation to the offspring, or deletions and duplications may occur through recombination between the normal allele and the inverted allele. The chance of a child receiving an unbalanced gamete is up to 50%. A child with this unbalanced rearrangement would be expected to have developmental delay and possible birth defects. This rearrangement can be in the form of deletion or duplication of the chromosomal segment in question. In the remaining off-springs, where the rearrangement remains balanced, there will be no expression of these problems. Alternately, the mother may pass on her normal number 22 chromosome, and the child will be normal [9]. 

CC, the older sibling (46, XY, rec (22) dup (22q) inv (22) (p13; q13.32) mat,) has no dysmorphic features, yet has significant developmental delay. The 22 chromosome duplications are very rare, and the ones cited in the literature have breakpoints on 22q12 rather than 22q13 [10]. 

JC, with monosomy of 22q13.3, has findings typical of 22q13.3 deletion syndrome patients including global developmental delay, generalized hypotonia, absent or severely delayed speech, and normal or accelerated growth. The true prevalence of 22q13.3 deletion may be underestimated because routine karyotyping may only detect a fraction of the cases [11]. When this condition is suspected based on clinical features or there is a history of mild GDD in an older sibling, early genetic testing should be instituted employing new and more assertive methods like genomic microarray and fluorescent in situ hybridization (FISH) [4]. Early genetic counseling should be considered. The extended family should also be considered for genetic testing. It has been reported that 22q13 deletions are most often paternal in origin [12]. It is worth noting that our case (JC) acquired the deletion from his mother.

The children received intensive physical, occupational, and speech therapies weekly. In addition, JC also received music therapy, picture exchange communication, and sign language instruction. These have been shown to be effective in autism [13]. It is believed that these interventions contributed to improving behavioral issues (autistic features) in JC and global developmental delay in both. To facilitate communication and decrease frustration within the family, all family members should be encouraged to learn sign language [Phelan, personal communication].

As is true for most chromosomal abnormalities, and as was done with the presented cases, individuals with 22q13.3 deletion or duplication should also undergo base-line investigations like renal ultrasound (for renal cysts and other structural problems), echocardiogram (for septal defects), EEG (for seizure activity), audiology tests (to r/o hearing deficits), and brain imaging studies (arachnoid cysts, etc.).

As these individuals are expected to have a reasonably normal life expectancy [Phelan, personal communication], they should receive the recommended health maintenance preventive services (PAP smears, colon cancer screening, prostate exam, cholesterol check, etc.). Some screening tests like colon cancer screening may need to be expedited, as there may be an increased predisposition towards colon cancer [14]. 

Amniocentesis for prenatal testing in pregnancies at increased risk for chromosomal abnormalities may not be diagnostic, as in JC's case [Dr. Grati, European Genetics Conf. 2008], and FISH, chorionic villus sampling, or micro-array testing should be considered, as they are more specific. 

Behavioral interventions and medication management such as atypical neuroleptics are often recommended to control behavior problems.

## Figures and Tables

**Figure 3:**